# Mucosal Microbiota and Metabolome along the Intestinal Tract Reveal a Location-Specific Relationship

**DOI:** 10.1128/mSystems.00055-20

**Published:** 2020-05-26

**Authors:** Ce Yuan, Melanie Graham, Christopher Staley, Subbaya Subramanian

**Affiliations:** a Bioinformatics and Computational Biology Program, University of Minnesota, Minneapolis, Minnesota, USA; b Department of Surgery, University of Minnesota, Minneapolis, Minnesota, USA; c Masonic Cancer Center, University of Minnesota, Minneapolis, Minnesota, USA; Institute for Systems Biology

**Keywords:** microbiota, metabolome, host-microbiota interactions, nonhuman primate, prebiotics

## Abstract

The intestinal microbiota is highly metabolically active and plays an important role in many metabolic processes absent from the human host. Altered microbiota metabolism has been linked to diseases such as obesity, cardiovascular disease, and colorectal cancer. However, there is a gap in the current knowledge on how the microbiota interact with its host in terms of metabolic interactions. Here, we performed an integrated analysis between the mucosa-associated microbiota and the mucosa metabolome in healthy, nonhuman primates to investigate these relationships. The microbiota composition was distinct at each tissue location, with variation by host individual also observed. Microbiota-metabolome dynamics were primarily driven by interactions in the distal colon. These interactions were strongly correlated with dietary component, indicating a possibility to modulate microbiota-metabolomic interactions using prebiotic strategies.

**IMPORTANCE** In a healthy colon, the microbiota produces a vast amount of metabolites that are essential to maintaining homeostasis in the colon microenvironment. In fact, these metabolites produced by the microbiota have been linked to diseases such as obesity, cardiovascular disease, and colorectal cancer. In this study, we used healthy nonhuman primate models to investigate the relationship between microbiota and tissue metabolites. We found that both microbiota and metabolites have location-specific signatures along the intestine. Most importantly, we found that metabolites from food sources correlate with multiple bacteria in different intestinal locations. Overall, this work presents a systems-level map of the association between the microbiota and the metabolites in healthy nonhuman primates, provides candidates for experimental validation, and suggests a possibility to regulate the gut microbiota through specific prebiotic combinations.

## Abstract (precis)

In a healthy colon, the microbiota produces a vast amount of metabolites that are essential to maintaining homeostasis in the colon microenvironment. In fact, these metabolites produced by the microbiota have been linked to diseases such as obesity, cardiovascular disease, and colorectal cancer. In this study, we used healthy nonhuman primate models to investigate the relationship between microbiota and tissue metabolites. We found that both microbiota and metabolites have location-specific signatures along the intestine. Most importantly, we found that metabolites from food sources correlate with multiple bacteria in different intestinal locations. Overall, this work presents a systems-level map of the association between the microbiota and the metabolites in healthy nonhuman primates, provides candidates for experimental validation, and suggests a possibility to regulate the gut microbiota through specific prebiotic combinations.

## INTRODUCTION

The human intestinal tract harbors trillions of microorganisms, termed the microbiota, which includes thousands of bacterial species (1). It has become evident that the gut microbiota is important in regulating and maintaining the health of the host and is implicated in many diseases, such as obesity and several cancers (2–6). Despite numerous studies indicating important roles of microbiota in diseases, most studies have primarily focused on variations in taxonomic composition of the microbiota. The underlying metabolic features associated with the host-microbiota interaction, however, still remain unclear for most diseases.

The gut microbiota produces a vast amount of metabolites. Some metabolites, such as vitamin B, vitamin K, bile acids, and short-chain fatty acids (SCFAs), are essential to maintaining homeostasis in the colon (7–9). The most direct and active metabolic interaction between the host and its microbiota is in the large intestine, and the vast majority (∼70%) of energy required by the normal colon epithelium comes from butyrate produced by the microbiota through fermentation of complex carbohydrates (10). Without a functional microbiota, the colon epithelia undergo autophagy and fail to maintain normal structure and function (11). Moreover, the metabolic interactions between the host and its microbiota have widespread implications throughout the body (8). For example, the obesity-associated microbiota has been shown to possess increased metabolic capability to harvest energy from food (12, 13), and the metabolism of l-carnitine by the gut microbiota has been shown to promote atherosclerosis (14). These studies suggest potential metabolic shifts of the microbiota, either in response to or responsible for the host metabolic state (13).

The mucosal host-microbiota metabolic interactions along a healthy human intestinal tract are largely unknown. Although the microbiota and metabolome variations along the intestinal tract have been investigated in rodents and other animals, the dietary and anatomical differences between humans and these animals render these data less informative for humans (15–20). Here, we investigated the microbiota and metabolome profiles along the intestinal tracts of healthy baboons (Papio anubis), a family of Old World monkeys. We collected tissue samples from the duodenum, jejunum, ileum, cecum, proximal colon, and distal colon. Amplicon sequencing of the 16S rRNA gene (16S-Seq) was used to identify the mucosal surface microbiota composition. We also performed untargeted metabolomics on the immediately adjacent tissues to profile the tissue metabolite contents.

## RESULTS

### Microbiota landscape along the nonhuman primate (NHP) intestinal tract.

**FIG 1:**
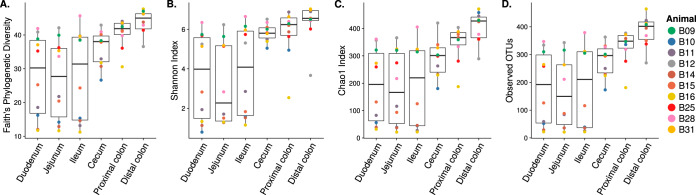
Microbiota alpha diversity along the intestinal tract. (A) Faith’s phylogenetic diversity, (B) Shannon index, (C) Chao1 index, and (D) number of observed OTUs.

**FIG 2:**
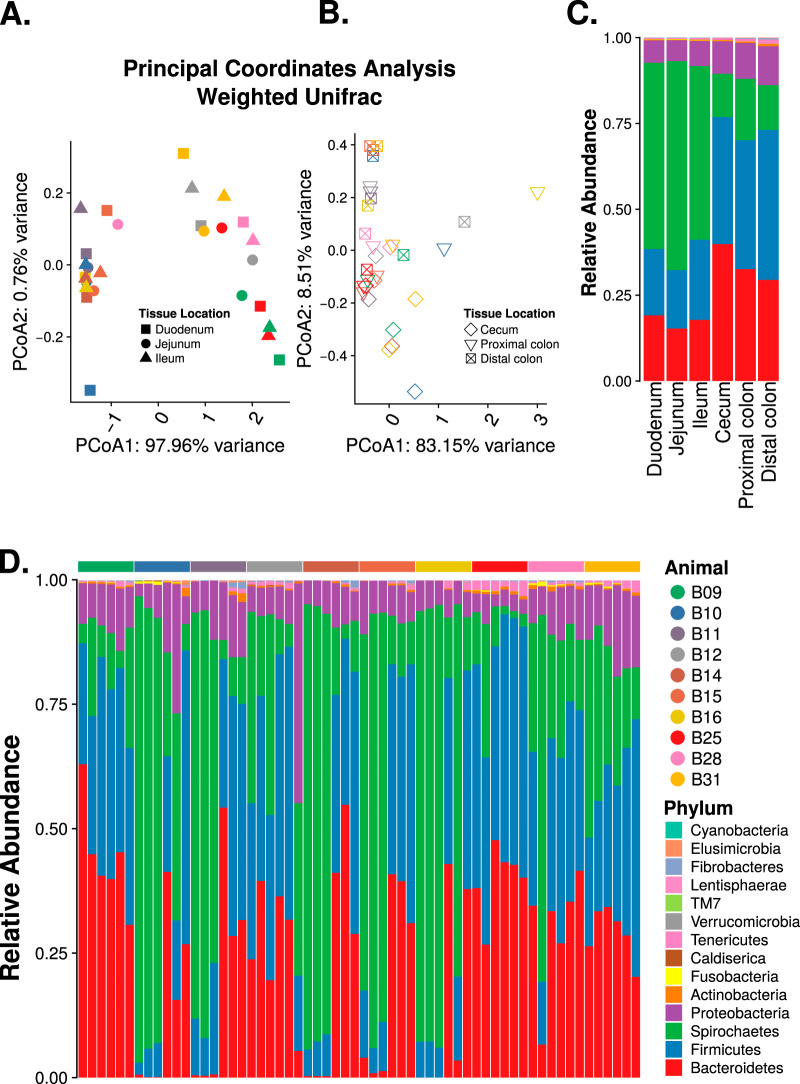
Microbiota along the nonhuman primate gastrointestinal tract. Weighted UniFrac principal-coordinate analysis (PCoA) of the upper (A) and lower (B) intestinal samples. Stacked bar plot of bacterial phyla showing the average relative abundance at each of the six tissue locations (C) and at each of the six tissue locations for each sample (D), in the order of duodenum, jejunum, ileum, cecum, proximal colon, and distal colon.

We first assessed the baboon intestinal-tissue-associated microbiota composition in 10 baboons using the 16S-Seq method. Baboons were between 7 and 16 years old and weighed 14 to 25 kg at the time of sample collection (see Table S1 in the supplemental material). We found that the small intestinal (duodenum, jejunum, and ileum) microbiota had significantly lower phylogenetic distance (*P* < 1 × 10^−5^, two-tailed *t* test; Fig. 1A), Shannon index (*P* < 1 × 10^−5^; Fig. 1B), Chao1 index (*P* < 1 × 10^−5^; Fig. 1C), and observed OTUs (*P* < 1 × 10^−5^; Fig. 1D) compared to the microbiota in the large intestine (cecum, proximal colon, and distal colon) (15, 20). The patterns of beta-diversity also differed between the upper and lower intestinal sites (Fig. 2A and B; see also Fig. S1 in the supplemental material). In the small intestine, differences in composition did not reflect different tissue sites (permutational multivariate analysis of variance [PERMANOVA] *R*^2^ = 0.02, *P* = 1), but significant compositional differences were observed between individual animals (*R*^2^ = 0.84, *P* < 0.0001; Fig. 2A). Conversely, in the large intestine, tissue-specific differences were observed (*R*^2^ = 0.16, *P* < 0.0005; Fig. 2B). However, compositional differences were more strongly driven by the individual host (*R*^2^ = 0.55, *P* < 0.0001). This suggests that both host and tissue locations can impact the mucosa-microbiota structure in the intestine (5).

10.1128/mSystems.00055-20.1 TABLE S1 Baboon metadata, differentially abundant metabolites in the small and large intestine, and Ingenuity pathway analysis. Download Table S1, XLSX file, 0.05 MB. Copyright © 2020 Yuan et al. 2020 Yuan et al. This content is distributed under the terms of the Creative Commons Attribution 4.0 International license.

10.1128/mSystems.00055-20.5 FIG S1 Principal-coordinate analysis (PCoA). (A) Weighted UniFrac PCoA showing PCoA1 versus PCoA3. Unweighted UniFrac PCoA showing PCoA1 versus PCoA2 (B) and PCoA1 versus PCoA3 (C). Download FIG S1, PDF file, 0.04 MB. Copyright © 2020 Yuan et al. 2020 Yuan et al. This content is distributed under the terms of the Creative Commons Attribution 4.0 International license.

**FIG 3:**
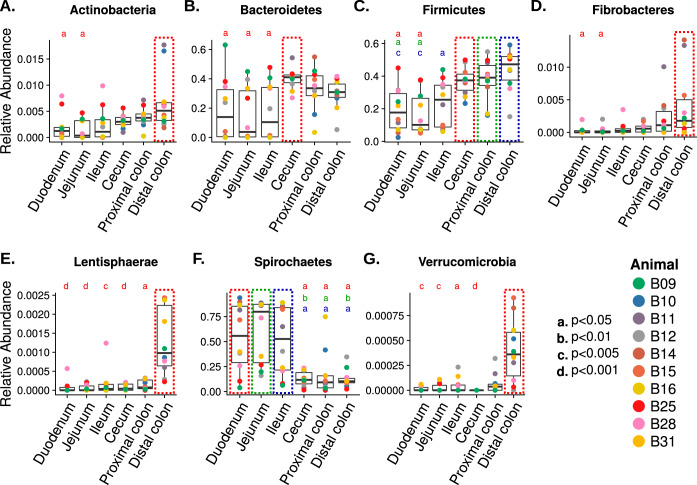
Box plot of bacterial phyla with differential abundances across different tissue sites. Seven bacterial phyla have differential abundance in tissue locations highlighted in the color-dotted box. Statistical significance are indicated by lowercase letters as follows: a, *P* < 0.05; b, *P* < 0.01; c, *P* < 0.005; d, *P* < 0.001.

**FIG 4:**
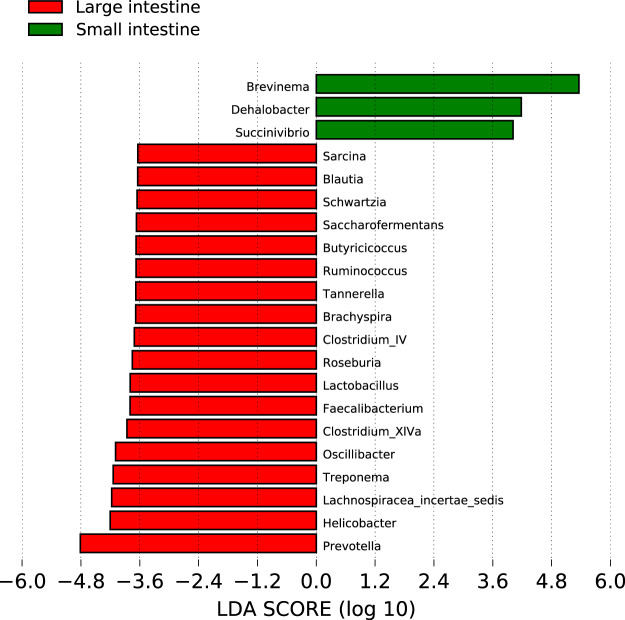
Linear discriminant analysis (LDA) effect size (LEfSe). Thirty-one bacterial taxa have an LDA score (log_10_) over 3.6. Bacteria with larger effect size in the large intestine are shown in red, and bacteria with larger effect size in the small intestine are shown in green.

The baboon intestinal tissue-associated microbiota was dominated by the bacterial phyla *Firmicutes*, *Bacteroidetes*, *Spirochaetes*, and *Proteobacteria*, independent of the tissue location (Fig. 2C and D). At the phylum level, seven taxa (*Actinobacteria*, *Bacteroidetes*, *Firmicutes*, *Fibrobacteres*, *Lentisphaerae*, *Spirochaetes*, and *Verrucomicrobia*) exhibited location-specific enrichment (Fig. 3; *P* value cutoff = 0.05, Kruskal-Wallis test with Dunn *post hoc* test). We next analyzed differences in operational taxonomic unit (OTU) composition to discover whether site-specific bacterial community signatures occurred. We performed linear discriminant analysis (LDA) effect size (LEfSe) and identified 21 taxa (at the genus level) that were characteristic of the small and large intestine (Fig. 4) (21). Of these 21 taxa, 3 taxa (*Brevinema*, *Dehalobacter*, and *Succinivibrio*) were characteristic of the small intestine.

### Metabolomic landscape along the baboon intestinal tract.

We then used Q Exactive LC-MS/MS (liquid chromatography coupled to tandem mass spectrometry) quadrupole Orbitrap (Thermo Scientific) to analyze the tissue metabolome composition in tissue samples immediately adjacent to the tissues used for 16S-Seq. A total of 3,395 compounds were present in at least two-thirds of all samples analyzed. After searching against the Human Metabolome Database (HMDB) and in-house libraries generated by the University of Minnesota Center for Mass Spectrometry and Proteomics, a total of 292 compounds were assigned putative identity. We focus on these compounds with assigned identities for further analysis.

We sought first to identify differential metabolites between the small and large intestine. We performed Wilcoxon rank sum test between metabolites of the small intestine (87) and large intestine (53) and identified 140 compounds with differential abundance. Consistent with previous studies in human and mouse samples, the small intestine contained more amino acids such as aspartic acid, alanine, tyrosine, valine, leucine, and isoleucine, as well as tauro-conjugated bile acids (22, 23). In the large intestine, there was more cholic acid and urobilin, in addition to more-complex metabolites. We then performed pathway analysis using the fold change differences of the differentially abundant compounds between the small and large intestine (Table S1). Curiously, we found that these compounds are involved in the upregulation of bacterial growth-related pathways (Fig. S2) in the small intestine. In the large intestine, amino acid uptake pathways (Fig. S3B) and cancer-related pathways (Fig. S3C) were upregulated.

10.1128/mSystems.00055-20.6 FIG S2 Ingenuity Pathway Analysis of differentially abundant metabolites. (A) Pathways related to growth of bacteria are activated in the small intestines. Pathways related to uptake of amino acids (B) and solid tumor (C) are activated in the large intestines. A blue line indicates activation, an orange line indicates inhibition, a yellow line indicates conflicting evidence, and a gray line indicates association. Download FIG S2, PDF file, 1.3 MB. Copyright © 2020 Yuan et al. 2020 Yuan et al. This content is distributed under the terms of the Creative Commons Attribution 4.0 International license.

10.1128/mSystems.00055-20.7 FIG S3 Tissue-specific Procrustes analysis. (A) Jejunum, (B) duodenum, (C) ileum, (D) cecum, (E) proximal colon, and (F) distal colon. Download FIG S3, PDF file, 0.03 MB. Copyright © 2020 Yuan et al. 2020 Yuan et al. This content is distributed under the terms of the Creative Commons Attribution 4.0 International license.

### Microbiota-metabolome interactions.

**FIG 5:**
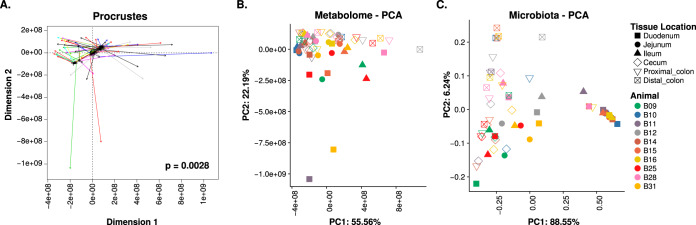
Microbiota-metabolome similarity. (A) Procrustes analysis of the microbiota principal-component analysis (PCA) against the metabolome PCA. Longer line lengths indicate lower within-sample similarities. PCA of the tissue metabolome (B) and microbiota (C).

**FIG 6:**
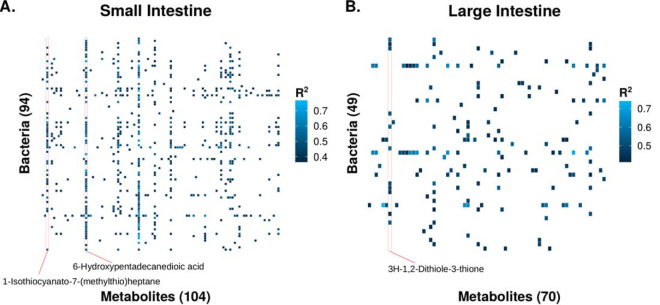
Heatmaps of significant microbiota-metabolite correlations of the small intestine (A) and large intestine (B).

To establish a global microbiota-metabolome relationship, we performed Procrustes analysis using the *vegan* package in R (Fig. 5A). Globally, we found a significant relatedness (*P* = 0.0028) between the microbiota and the metabolome. Interestingly, this relatedness was driven by the ileum (*P* = 0.042) and distal colon (*P* = 0.037; Fig. S3). We then analyzed microbiota-metabolite relationships using Spearman’s ranked correlation on the metabolites with assigned identity and abundances of bacterial genera. This includes 595 significant (*q* < 0.05, false discovery rate-adjusted *P* value) interactions in the small intestine and 166 in the large intestine (Table 1; Table S2). Additionally, we observed that the correlation network in the small intestine was more interconnected than that in the large intestine (Fig. 6). One explanation is that the large intestine harbors more bacterial species than the small intestine; thus, there could be more functional redundancies in the large intestine, with fewer correlations at greater taxonomic resolution. Interestingly, in both networks, the levels of most metabolites were correlated with only a few bacterial taxa, and such correlations tended to be in the same direction (Table 1; Fig. 6). However, a bacterial taxon tended to correlate with many metabolites in different directions. Although the current data do not demonstrate any causal relationships among the mucosal microbiota and metabolites, it nevertheless warrants further investigation.

**TABLE 1:** Summary of microbiota-metabolome interactions

Location	No. of metabolites with >5 bacterial interactions	No. of bacteria with >5 metabolite interactions	No. of significant interaction pairs
Small intestine	26	49	595
Large intestine	8	8	166

10.1128/mSystems.00055-20.2 TABLE S2 Spearman’s correlation results for microbiota-metabolome interactions in the small and large intestine. Download Table S2, XLSX file, 0.04 MB. Copyright © 2020 Yuan et al. 2020 Yuan et al. This content is distributed under the terms of the Creative Commons Attribution 4.0 International license.

### Dietary enrichment shapes intestinal tract microbiota-metabolite interactions.

We further sought to determine the potential origin of the metabolites that were highly correlated with bacterial taxa. These metabolites corresponded to metabolites commonly found in several vegetables. 6-Hydroxypentadecanedioic acid and 1-isothiocyanato-7-(methylthio)heptane had 56 and 31 significant correlations in the small intestine, respectively (Fig. 6A). 3H-1,2-Dithiole-3-thione had 11 significant correlations in the large intestine (Fig. 6B). Surprisingly, all three compounds are commonly found in *Brassica* vegetables, which were fed to the animals as a part of the normal dietary enrichment. All bacteria significantly correlated with 6-hydroxypentadecanedioic acid in the small intestine are positively correlated with this compound (Table S2).They include *Clostridium* XlVa, *Ruminococcus*, *Faecalibacterium*, and *Lactobacillus*, all of which have shown health benefits in humans (24, 25). This suggests that 6-hydroxypentadecanedioic acid may have potential prebiotic effects. Due to the potential health benefits associated with eating *Brassica* vegetables, this finding warrants additional investigation.

## DISCUSSION

Currently, there is limited knowledge of the microbiota composition along different sections of the intestinal tract in either human or nonhuman primate (NHP) samples (26, 27). Studies of human subjects usually require prior bowel preparation, which has been shown to alter the microbiota (28). In this study, we collected tissue samples from healthy NHPs without prior bowel preparation, thus providing an unaltered view of the healthy microbiota. Previous studies have analyzed the intestinal tract microbiota compositions in mice, chickens, dogs, cows, and horses (15–19), among others. However, due to the anatomical differences, in addition to the dietary and genetic differences, these animals may have different microbiota along the intestinal tract.

Perhaps not surprisingly, the baboon microbiota composition is more similar to that observed in human intestinal tissue-associated microbiota and dissimilar to that observed in mouse fecal samples (6). Similar to a previous human study which examined the microbiota composition using small and large intestinal biopsy samples (20), we found several microbiota differences along the intestinal tract at the phylum level. Additionally, we found lower alpha-diversity in the small intestine, while Stearns et al. (20) did not. One plausible explanation is the previous study collected biopsy samples after the patients had undergone bowel preparation, and this may affect the microbiota composition. Indeed, the fecal samples collected prior to bowel preparation had very different microbiota composition compared to the colon tissue samples.

Similar to previous reports in humans, we found variations in the microbiota composition between different NHP subjects. In addition, we found that the microbiota composition along the intestinal tract is also influenced by the host. Previous studies suggest that this variation between individuals can be attributed to factors such as genetics, dietary preferences, and other factors (5, 29, 30). In this study, differences in baboon age and weight may have further contributed to interindividual differences. The alpha-diversity differences observed along the intestinal tract sections were likely due to changes in the microbial concentration gradient, where the small intestine harbors fewer bacteria due to the high-pH environment. It is not surprising that the distal colon and cecum harbor more distinct bacterial taxa than other locations. Previous studies have shown that both the distal colon and cecum are where most bacterial fermentation takes place, although we found no discernible differences in the predicted metagenome of the microbiota (31).

In this study, we performed untargeted metabolomics on the intestinal tissues. Although we were able to identify more than 3,395 entities, we were able to assign identities to only 292 compounds. This lack of positive identification is mainly due to the lack of available databases. It is conceivable that we will be able to extract additional information from the current data in the future using improved databases, further strengthening the current research. Nevertheless, using the most current database, we found that the metabolomic profiles showed enriched cancer-related pathways in the large intestine. We think this observation suggests that the large intestinal metabolic microenvironment may better support tumor growth compared to the small intestine. This hypothesis is supported by the low incidence of small intestinal tumors in humans. However, a major caveat of the current research was our inability to distinguish between the metabolic contribution from the host and the microbiota. Thus, whether differences in metabolite profiles between the small and large intestine were primarily driven by the microbiota is yet to be determined. A previous study comparing the tissue-level metabolome between conventional and germfree mice showed that the microbiota contribute to various metabolomic differences along the intestinal tract. However, whether this difference is due to changes in microbial metabolism or host metabolism is unclear (23). Future studies should aim to separate metabolites originated from the host, microbiota, or food source.

Interestingly, our analysis also found that 6-hydroxypentadecanedioic acid, 1-isothiocyanato-7-(methylthio)heptane, and 3H-1,2-dithiole-3-thione, compounds commonly found in *Brassica* vegetables, were correlated with higher levels of several potentially beneficial bacteria. Notably, 3H-1,2-dithiole-3-thione has been previously shown as a potent antioxidant and potential chemopreventive agent, by targeting the transcription factor NRF2 (32). This may suggest a potential prebiotic effect of these compounds. Moreover, these compounds show location-specific correlations with microbiota, which may suggest a potential strategy to target beneficial bacteria in different intestinal locations. One explanation for the location-specific correlations is the differences of absorption of these compounds at different locations of the intestine, which can lead to different metabolite concentrations in the intestinal lumen. However, since the current study did not include controlled feeding, we are unable to ascertain the exact role of these compounds in modulating the microbiota.

In the present study, we report the host-microbiota interactions along the healthy nonhuman primate lower gastrointestinal tract. Our study provided a global view of the microbiota landscape of healthy NHPs. Our analysis suggests an intricate global relationship between the microbiota and metabolites along the intestinal tract. Importantly, we found that dietary components may be a means to modify microbiota composition at specific sites throughout the intestinal tract, suggesting potential targeted use as prebiotic therapeutics. Further study will be necessary to evaluate specific diet-microbiota-metabolomic interactions and the potential to use metabolites as microbiota-directed therapeutics.

## MATERIALS AND METHODS

The tissue samples were collected via tissue sharing postmortem, which is exempt from Institutional Animal Care and Use Committee (IACUC) review. The cohort included 10 adult purpose-bred female olive baboons (Papio anubis) modeling anterior cruciate ligament (ACL) injury and subsequent repair using regenerative medicine techniques. The animals were between 6.5 and 15.6 years old (median, 9.3 years) and weighed between 14.4 and 24.9 kg (median, 20.1 kg). They were housed in pairs or housed in protected contact with compatible conspecifics. Baboons had free access to water and were fed identical diets that included biscuits (Harlan primate diet 2055C; Harlan Teklad) based on body weight and daily enrichment with fresh fruits, vegetables, grains, beans, nuts, and a multivitamin preparation. Semiannual veterinary physical examinations were performed on all animals. Animals participated in an environmental enrichment program designed to encourage sensory engagement, enhance foraging behavior and novelty seeking, promote mental stimulation, increase exploration and play and activity levels, and strengthen social behaviors, providing opportunities for animals to increase time spent on species-typical behaviors. Baboons were trained to cooperate with medical procedures, including hand feeding and drinking, shifting into transport cages for sedation, and targeting or presentation for examination. Animals were euthanized via barbiturate overdose (Beuthanasia-D ≥86 mg/kg of body weight intravenously), and tissue procurement was performed postmortem. No oral medications were used for at least 6 months prior to tissue collection. Tissue sections (approximately 1 cm by 1 cm) from six different sites that included the duodenum, jejunum, ileum, cecum, proximal colon, and distal colon were collected from each animal, a total of 60 samples, using clean technique, snap-frozen in liquid nitrogen, and then stored at –80°C.

### 16S-Seq and sequence analysis.

Total DNA was extracted from approximately 250 mg of tissue using DNeasy PowerSoil kit (catalog no. 12888; Qiagen, Valencia, CA) following the standard protocol. Sequencing libraries were created by the Mayo Clinic Genome Analysis Core (Rochester, MN). Briefly, the V3-V5 region of the 16S rRNA gene was amplified with multiplexing barcodes using PCR (V3-341F, TCGTCGGCAGCGTCAGATGTGTATAAGAGACAGCCTACGGGAGGCAGCAG; V5-926R, GTCTCGTGGGCTCGGAGATGTGTATAAGAGACAGCCGTCAATTCMTTTRAGT). The libraries were then pooled and size selected between 700 and 730 bp using a LabChip XT (PerkinElmer, Waltham, MA). Sequencing was performed on a single lane of a MiSeq sequencer (Illumina) using paired-end mode. On average, 64,937 quality reads (between 9,901 and 118,288) were generated per library. The sequencing results were analyzed using the IM-TORNADO2 pipeline (33). Alpha- and beta-diversity metrics were analyzed using QIIME v1.9.1 (34). The unfiltered OTU table is available in Table S2 in the supplemental material. Linear discriminant analysis of effect size (LEfSe) was used to determine differences in the relative abundances of taxa among tissue sites (21).

The beta-diversity between tissue locations was analyzed by performing principal-coordinate analysis (PCoA) using both weighted and unweighted UniFrac distance metrics. The unweighted UniFrac distance considers only the presence and absence of a certain OTU, while the weighted UniFrac distance will consider the abundance; thus, these metrics can give an overview of the microbial structure differences of different tissue locations (35–37).

### Metabolite extraction.

Metabolites were extracted from the immediately adjacent tissue that was used to generate 16S-Seq. There was an insufficient amount of duodenum tissue from animal B09 to perform untargeted metabolomics, so it was not analyzed. Approximately 15 mg of tissue was used to extract metabolites. The tissues were first ground into fine powder using CryoGrinder (OPS Diagnostics) on dry ice. The tissues were then suspended in 20 μl of 80% methanol per 1 mg of tissue weight. The mixture was then homogenized using a probe sonicator at 10% amplitude for 15 s, with 1-min rest on ice after 5 s of sonication. The sonicated samples were then centrifuged at 14,000 × *g* for 10 min at 4°C. The supernatant from the centrifugation contained the metabolites and was saved at –80°C before drying. The tissue pellets were then further processed for additional metabolite extraction. They were first suspended in 10 μl of 80% methanol per 1 mg of original tissue weight and sent through high-pressure cycling on a Barocycler NEP2320 (Pressure Biosciences). The high-pressure cycling protocol includes 60 cycles of 20 s of 35,000 lb/in^2^ pressure, followed by 10 s of 0 lb/in^2^ for at 4°C. After pressure cycling, the samples were again centrifuged at 14,000 × *g* for 10 min at 4°C, and the supernatants were pooled with the previously extracted metabolites. Finally, the metabolites were dried under a nitrogen stream.

### Untargeted metabolomics.

The dried metabolites were first suspended in 15 μl of 0.1% formic acid per 1 mg of the original tissue weight. The suspensions were then separated for analysis using a C_18_ reverse-phase column and hydrophilic interaction liquid chromatography (HILIC) column. The reverse-phase analysis results in separation of larger nonpolar molecules such as steroid-like compounds, certain amino acids, phospholipids, and other lipids, while the HILIC analysis separates hydrophilic compounds such as amino acids and member of the citric acid cycle and glycolysis pathways. The samples were analyzed using reverse-phase positive mode (nonpolar interaction) separation and HILIC analysis (polar interaction) separation before analysis with Q Exactive LC-MS/MS quadrupole Orbitrap (Thermo Scientific). The reverse-phase analysis was performed in positive mode ionization with an additional proton (+1.0073) added. For HILIC analysis, the negative ionization mode was used with one additional proton (−1.0073) removed. Since salts are present, compounds may occasionally form as a sodium salt (neutral mass plus 21.9944) for the positive mode or as a chloride salt (neutral mass plus 34.9688) for the negative mode. Samples were loaded and analyzed in random order, and quality control samples were analyzed at regular intervals to eliminate extraneous signals. The untargeted metabolomics were performed by the University of Minnesota Center for Mass Spectrometry and Proteomics.

### Metabolomic data analysis.

The data were processed using Progenesis QI software (Thermo). The software first aligns all the features obtained in all the runs and then assigns intensity measures for features found in all the runs. The raw data were further processed by filtering for fidelity of individual feature detection using the quality control samples. Only features with a coefficient of variation (CV) of less than 10% overall quality control samples were accepted. Features showing high intensity in background samples relative to the quality control samples and features not present in at least 67% of all samples were removed from analysis per the U.S. Food and Drug Administration recommendation. Each feature is uniquely identified with the mass-to-charge ratio (*m/z*) and the elution time from the column. Features were then assigned to metabolites identified by searching the Human Metabolome DataBase (HMDB) and using databases developed by the University of Minnesota (Table S3). Pathway analysis was performed using Ingenuity Pathway Analysis (IPA).

10.1128/mSystems.00055-20.3 TABLE S3 Unfiltered OTU table used for analysis. Download Table S3, TXT file, 0.2 MB. Copyright © 2020 Yuan et al. 2020 Yuan et al. This content is distributed under the terms of the Creative Commons Attribution 4.0 International license.

### Microbiome-metabolome correlation analysis.

All analyses were performed in R v3.4.4 unless otherwise noted. The Spearman’s ranked correlation test with false-discovery rate (FDR) adjustment was used to test the microbiome-metabolome correlation (38). The microbiome OTU data and metabolomic data were first combined and filtered to remove low-abundance OTUs and metabolites (appearing in less than 50% of samples). The Spearman’s ranked correlation test was calculated using the *cor.test* function. The *P* values were then adjusted using the *p.adjust* function before filtering for significant correlations. PERMANOVA was performed using *adonis* function with Bray-Curtis distance and 999 permutations. Procrustes analysis was performed using the *procrustes* function of the *vegan* package in R with principal-component analyses of both the microbiome and metabolome using default options (38).

### Availability of data and material.

All data generated or analyzed during this study and animal information are included in this published article and its supplemental material files. The 16S-Seq data are available through the National Center for Biotechnology Information (NCBI) BioProject under accession number PRJNA623075.

10.1128/mSystems.00055-20.4 TABLE S4 Unfiltered untargeted metabolomic data used for analysis. Download Table S4, XLSX file, 2.1 MB. Copyright © 2020 Yuan et al. 2020 Yuan et al. This content is distributed under the terms of the Creative Commons Attribution 4.0 International license.

## References

[1] Blaut M, Clavel T 2007 Metabolic diversity of the intestinal microbiota: implications for health and disease. J Nutr 137:751S–755S. doi:10.1093/jn/137.3.751S. 17311972

[2] Abdollahi-Roodsaz S, Abramson SB, Scher JU 2016 The metabolic role of the gut microbiota in health and rheumatic disease: mechanisms and interventions. Nat Rev Rheumatol 12:446–455. doi:10.1038/nrrheum.2016.68. 27256713

[3] Belkaid Y, Hand TW 2014 Role of the microbiota in immunity and inflammation. Cell 157:121–141. doi:10.1016/j.cell.2014.03.011. 24679531 PMC4056765

[4] Vangay P, Ward T, Gerber JS, Knights D 2015 Antibiotics, pediatric dysbiosis, and disease. Cell Host Microbe 17:553–564. doi:10.1016/j.chom.2015.04.006. 25974298 PMC5555213

[5] Yuan C, Burns MB, Subramanian S, Blekhman R 2018 Interaction between host microRNAs and the gut microbiota in colorectal cancer. mSystems 3:e00205-17. doi:10.1128/mSystems.00205-17. 29795787 PMC5954203

[6] Burns MB, Lynch J, Starr TK, Knights D, Blekhman R 2015 Virulence genes are a signature of the microbiome in the colorectal tumor microenvironment. Genome Med 7:55. doi:10.1186/s13073-015-0177-8. 26170900 PMC4499914

[7] Louis P, Hold GL, Flint HJ 2014 The gut microbiota, bacterial metabolites and colorectal cancer. Nat Rev Microbiol 12:661–672. doi:10.1038/nrmicro3344. 25198138

[8] Nicholson JK, Holmes E, Kinross J, Burcelin R, Gibson G, Jia W, Pettersson S 2012 Host-gut microbiota metabolic interactions. Science 336:1262–1267. doi:10.1126/science.1223813. 22674330

[9] Said HM, Mohammed ZM 2006 Intestinal absorption of water-soluble vitamins: an update. Curr Opin Gastroenterol 22:140–146. doi:10.1097/01.mog.0000203870.22706.52. 16462170

[10] Scheppach W 1994 Effects of short chain fatty acids on gut morphology and function. Gut 35:S35–S38. doi:10.1136/gut.35.1_suppl.s35. 8125387 PMC1378144

[11] Donohoe DR, Garge N, Zhang X, Sun W, O’Connell TM, Bunger MK, Bultman SJ 2011 The microbiome and butyrate regulate energy metabolism and autophagy in the mammalian colon. Cell Metab 13:517–526. doi:10.1016/j.cmet.2011.02.018. 21531334 PMC3099420

[12] Turnbaugh PJ, Ley RE, Mahowald MA, Magrini V, Mardis ER, Gordon JI 2006 An obesity-associated gut microbiome with increased capacity for energy harvest. Nature 444:1027–1031. doi:10.1038/nature05414. 17183312

[13] Samuel BS, Hansen EE, Manchester JK, Coutinho PM, Henrissat B, Fulton R, Latreille P, Kim K, Wilson RK, Gordon JI 2007 Genomic and metabolic adaptations of Methanobrevibacter smithii to the human gut. Proc Natl Acad Sci U S A 104:10643–10648. doi:10.1073/pnas.0704189104. 17563350 PMC1890564

[14] Koeth RA, Wang Z, Levison BS, Buffa JA, Org E, Sheehy BT, Britt EB, Fu X, Wu Y, Li L, Smith JD, DiDonato JA, Chen J, Li H, Wu GD, Lewis JD, Warrier M, Brown JM, Krauss RM, Tang WHW, Bushman FD, Lusis AJ, Hazen SL 2013 Intestinal microbiota metabolism of L-carnitine, a nutrient in red meat, promotes atherosclerosis. Nat Med 19:576–585. doi:10.1038/nm.3145. 23563705 PMC3650111

[15] Gu S, Chen D, Zhang J-N, Lv X, Wang K, Duan L-P, Nie Y, Wu X-L 2013 Bacterial community mapping of the mouse gastrointestinal tract. PLoS One 8:e74957. doi:10.1371/journal.pone.0074957. 24116019 PMC3792069

[16] Stanley D, Hughes RJ, Moore RJ 2014 Microbiota of the chicken gastrointestinal tract: influence on health, productivity and disease. Appl Microbiol Biotechnol 98:4301–4310. doi:10.1007/s00253-014-5646-2. 24643736

[17] Gong J, Si W, Forster RJ, Huang R, Yu H, Yin Y, Yang C, Han Y 2007 16S rRNA gene-based analysis of mucosa-associated bacterial community and phylogeny in the chicken gastrointestinal tracts: from crops to ceca. FEMS Microbiol Ecol 59:147–157. doi:10.1111/j.1574-6941.2006.00193.x. 17233749

[18] Suchodolski JS, Camacho J, Steiner JM 2008 Analysis of bacterial diversity in the canine duodenum, jejunum, ileum, and colon by comparative 16S rRNA gene analysis. FEMS Microbiol Ecol 66:567–578. doi:10.1111/j.1574-6941.2008.00521.x. 18557939

[19] Mao S, Zhang M, Liu J, Zhu W 2015 Characterising the bacterial microbiota across the gastrointestinal tracts of dairy cattle: membership and potential function. Sci Rep 5:16116. doi:10.1038/srep16116. 26527325 PMC4630781

[20] Stearns JC, Lynch MDJ, Senadheera DB, Tenenbaum HC, Goldberg MB, Cvitkovitch DG, Croitoru K, Moreno-Hagelsieb G, Neufeld JD 2011 Bacterial biogeography of the human digestive tract. Sci Rep 1:170. doi:10.1038/srep00170. 22355685 PMC3240969

[21] Segata N, Izard J, Waldron L, Gevers D, Miropolsky L, Garrett WS, Huttenhower C 2011 Metagenomic biomarker discovery and explanation. Genome Biol 12:R60. doi:10.1186/gb-2011-12-6-r60. 21702898 PMC3218848

[22] Wang Y, Holmes E, Comelli EM, Fotopoulos G, Dorta G, Tang H, Rantalainen MJ, Lindon JC, Corthésy-Theulaz IE, Fay LB, Kochhar S, Nicholson JK 2007 Topographical variation in metabolic signatures of human gastrointestinal biopsies revealed by high-resolution magic-angle spinning 1H NMR spectroscopy. J Proteome Res 6:3944–3951. doi:10.1021/pr0702565. 17711324

[23] Claus SP, Tsang TM, Wang Y, Cloarec O, Skordi E, Martin F-P, Rezzi S, Ross A, Kochhar S, Holmes E, Nicholson JK 2008 Systemic multicompartmental effects of the gut microbiome on mouse metabolic phenotypes. Mol Syst Biol 4:219. doi:10.1038/msb.2008.56. 18854818 PMC2583082

[24] Kong F, Hua Y, Zeng B, Ning R, Li Y, Zhao J 2016 Gut microbiota signatures of longevity. Curr Biol 26:R832–R833. doi:10.1016/j.cub.2016.08.015. 27676296

[25] Gorbach SL 2000 Probiotics and gastrointestinal health. Am J Gastroenterol 95:S2–S4. doi:10.1016/s0002-9270(99)00806-0. 10634218

[26] Eckburg PB, Bik EM, Bernstein CN, Purdom E, Dethlefsen L, Sargent M, Gill SR, Nelson KE, Relman DA 2005 Diversity of the human intestinal microbial flora. Science 308:1635–1638. doi:10.1126/science.1110591. 15831718 PMC1395357

[27] McKenna P, Hoffmann C, Minkah N, Aye PP, Lackner A, Liu Z, Lozupone CA, Hamady M, Knight R, Bushman FD 2008 The macaque gut microbiome in health, lentiviral infection, and chronic enterocolitis. PLoS Pathog 4:e20. doi:10.1371/journal.ppat.0040020. 18248093 PMC2222957

[28] Harrell L, Wang Y, Antonopoulos D, Young V, Lichtenstein L, Huang Y, Hanauer S, Chang E 2012 Standard colonic lavage alters the natural state of mucosal-associated microbiota in the human colon. PLoS One 7:e32545. doi:10.1371/journal.pone.0032545. 22389708 PMC3289660

[29] Blekhman R, Goodrich JK, Huang K, Sun Q, Bukowski R, Bell JT, Spector TD, Keinan A, Ley RE, Gevers D, Clark AG 2015 Host genetic variation impacts microbiome composition across human body sites. Genome Biol 16:191. doi:10.1186/s13059-015-0759-1. 26374288 PMC4570153

[30] Blekhman R, Perry GH, Shahbaz S, Fiehn O, Clark AG, Gilad Y 2014 Comparative metabolomics in primates reveals the effects of diet and gene regulatory variation on metabolic divergence. Sci Rep 4:5809. doi:10.1038/srep05809. 25069065 PMC4894427

[31] Martin F-P, Wang Y, Yap IKS, Sprenger N, Lindon JC, Rezzi S, Kochhar S, Holmes E, Nicholson JK 2009 Topographical variation in murine intestinal metabolic profiles in relation to microbiome speciation and functional ecological activity. J Proteome Res 8:3464–3474. doi:10.1021/pr900099x. 19492798

[32] Kwak M-K, Kensler TW 2010 Targeting NRF2 signaling for cancer chemoprevention. Toxicol Appl Pharmacol 244:66–76. doi:10.1016/j.taap.2009.08.028. 19732782 PMC3584341

[33] Jeraldo P, Kalari K, Chen X, Bhavsar J, Mangalam A, White B, Nelson H, Kocher J-P, Chia N 2014 IM-TORNADO: a tool for comparison of 16S reads from paired-end libraries. PLoS One 9:e114804. doi:10.1371/journal.pone.0114804. 25506826 PMC4266640

[34] Caporaso JG, Kuczynski J, Stombaugh J, Bittinger K, Bushman FD, Costello EK, Fierer N, Peña AG, Goodrich JK, Gordon JI, Huttley GA, Kelley ST, Knights D, Koenig JE, Ley RE, Lozupone CA, McDonald D, Muegge BD, Pirrung M, Reeder J, Sevinsky JR, Turnbaugh PJ, Walters WA, Widmann J, Yatsunenko T, Zaneveld J, Knight R 2010 QIIME allows analysis of high-throughput community sequencing data. Nat Methods 7:335–336. doi:10.1038/nmeth.f.303. 20383131 PMC3156573

[35] Lozupone CA, Hamady M, Kelley ST, Knight R 2007 Quantitative and qualitative beta diversity measures lead to different insights into factors that structure microbial communities. Appl Environ Microbiol 73:1576–1585. doi:10.1128/AEM.01996-06. 17220268 PMC1828774

[36] Bray JR, Curtis JT 1957 An ordination of the upland forest communities of southern Wisconsin. Ecol Monogr 27:325–349. doi:10.2307/1942268.

[37] Clarke KR 1993 Non-parametric multivariate analyses of changes in community structure. Austral Ecol 18:117–143. doi:10.1111/j.1442-9993.1993.tb00438.x.

[38] McHardy IH, Goudarzi M, Tong M, Ruegger PM, Schwager E, Weger JR, Graeber TG, Sonnenburg JL, Horvath S, Huttenhower C, McGovern DP, Fornace AJ, Borneman J, Braun J 2013 Integrative analysis of the microbiome and metabolome of the human intestinal mucosal surface reveals exquisite inter-relationships. Microbiome 1:17. doi:10.1186/2049-2618-1-17. 24450808 PMC3971612

