# Supplementary figures and images for: Mucosal Microbiota and Metabolome along the Intestinal Tract Reveal a Location-Specific Relationship
Source: mSystems. 2020 May 26;5(3):e00055-20. doi: 10.1128/mSystems.00055-20 (PMC7253361; doi:10.1128/mSystems.00055-20)

A.

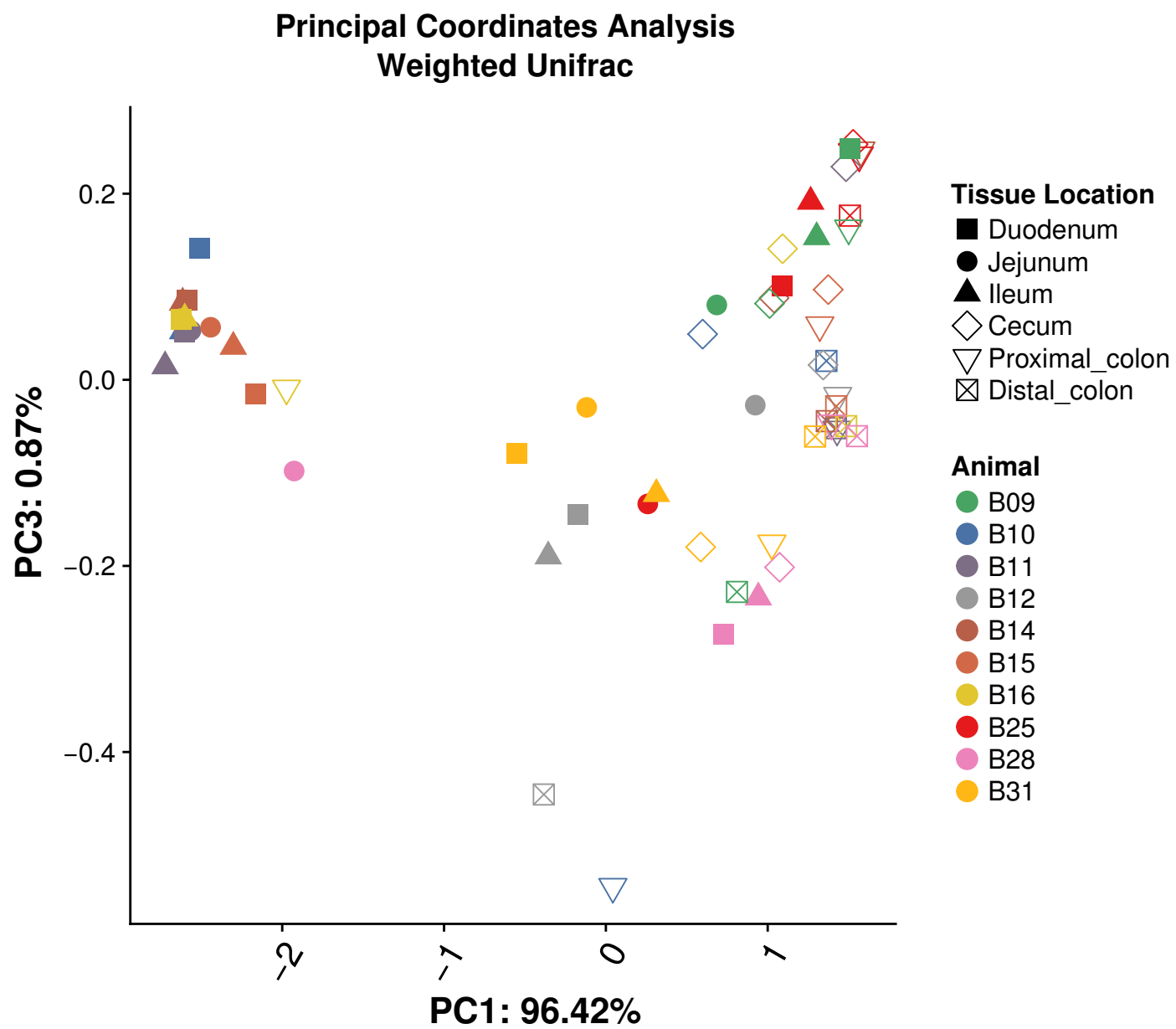

B.

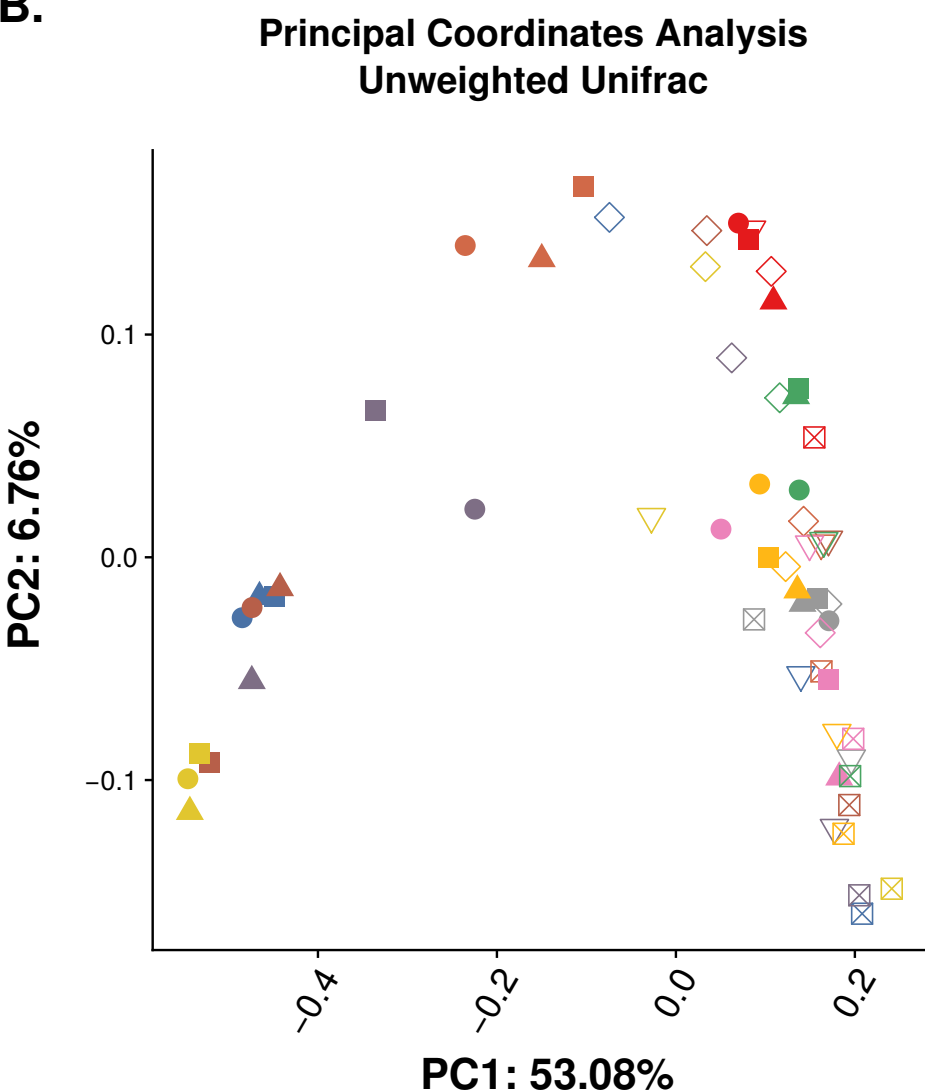

C.

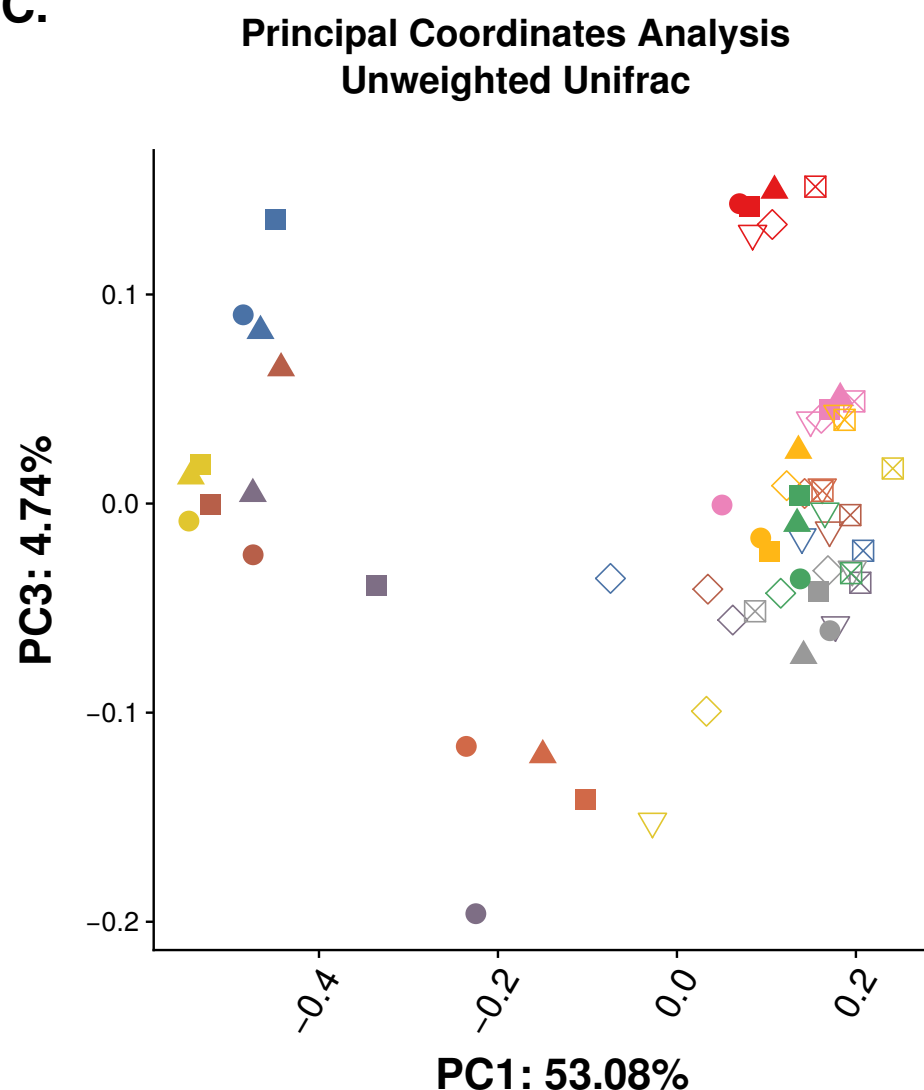

Supplement: FIG S1 [file mSystems.00055-20-sf001.pdf]

A.

Growth of bacteria 1

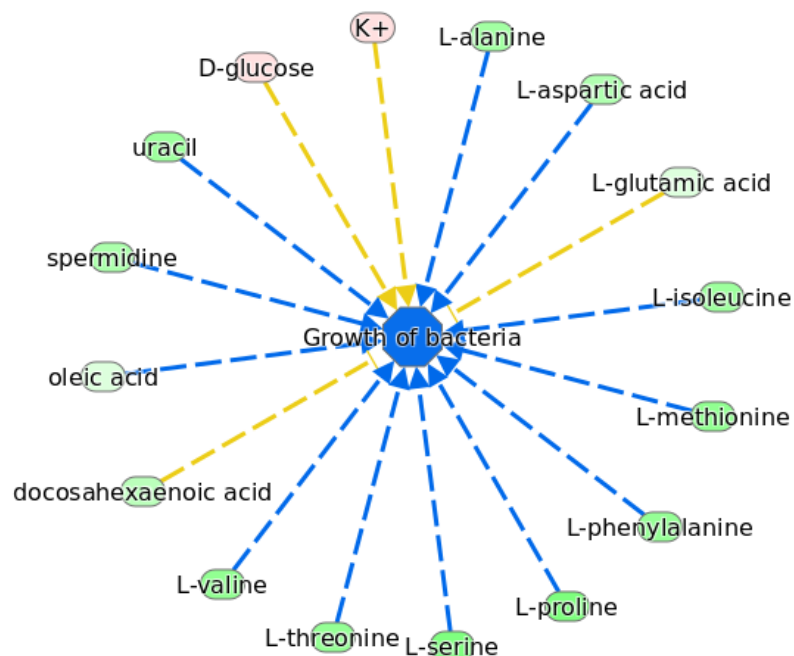

B.

Uptake of amino acids 3

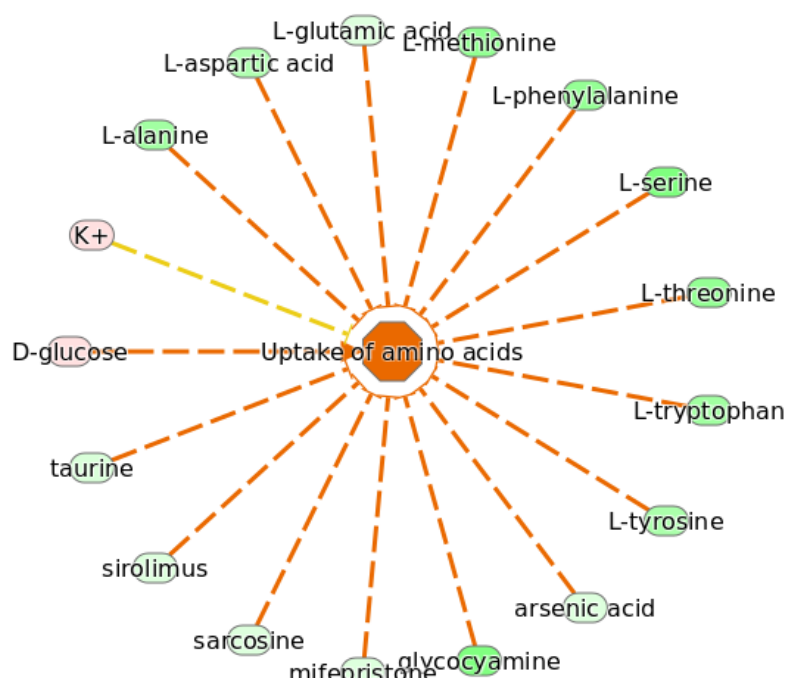

C.

Solid tumor 4

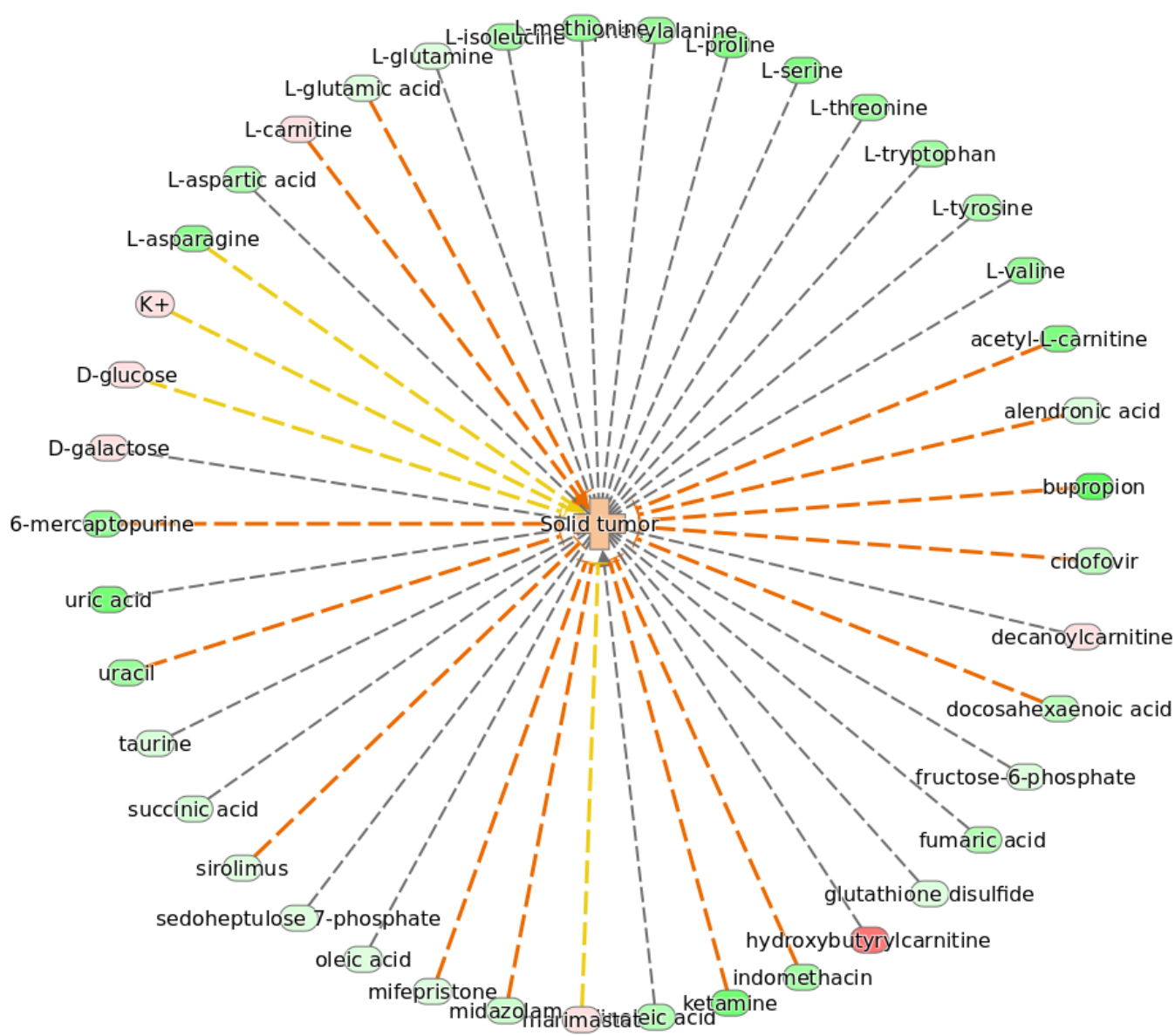

Supplement: FIG S2 [file mSystems.00055-20-sf002.pdf]
