# Supplementary material for: Mucosal Microbiota and Metabolome along the Intestinal Tract Reveal a Location-Specific Relationship
Source: mSystems. 2020 May 26;5(3):e00055-20. doi: 10.1128/mSystems.00055-20 (PMC7253361; doi:10.1128/mSystems.00055-20)
Supplement: FIG S3 [file mSystems.00055-20-sf003.pdf]

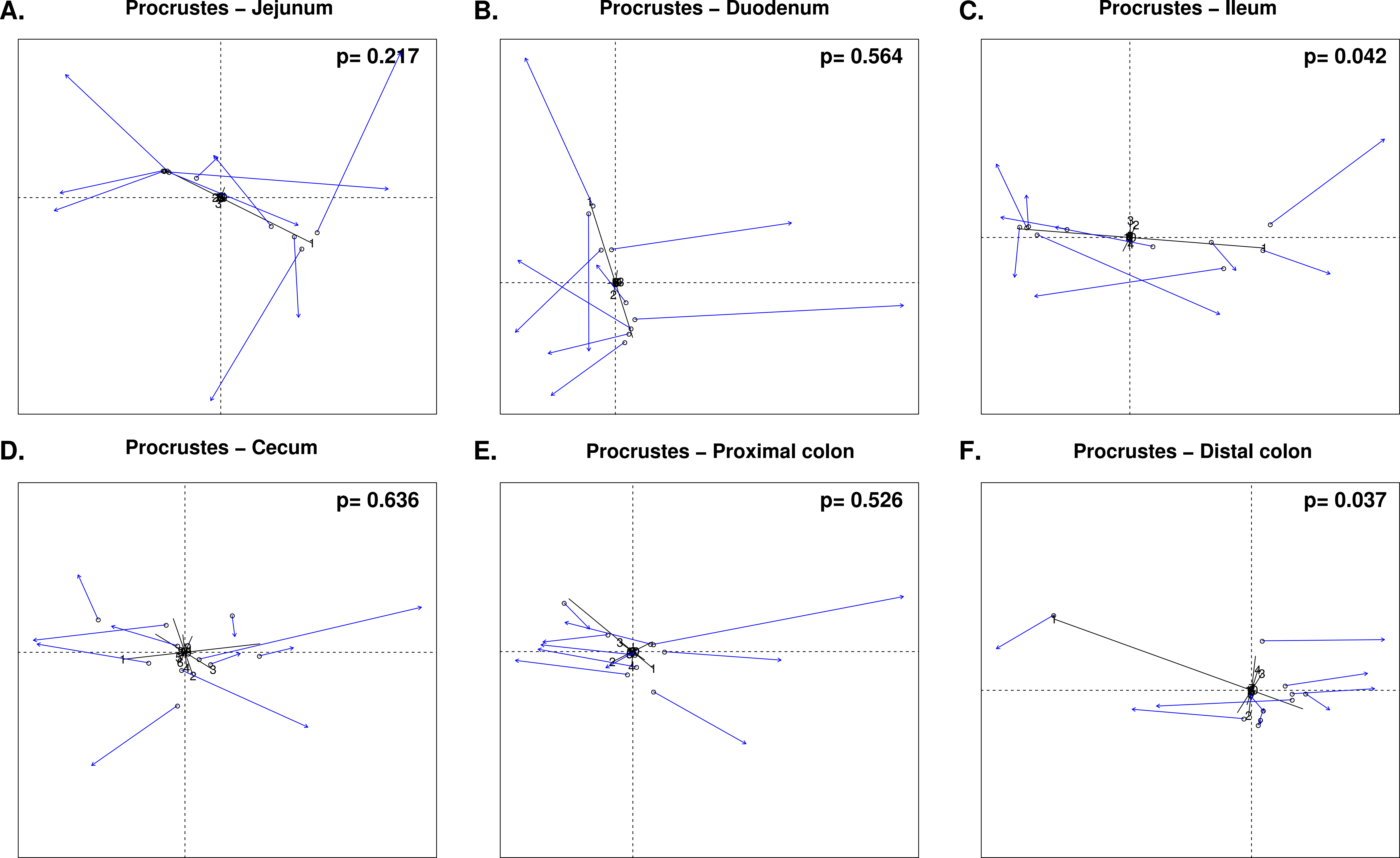
A.
Procrustes – Jejunum
p= 0.217
B.
Procrustes – Duodenum
p= 0.564
C.
Procrustes – Ileum
p= 0.042
D.
Procrustes – Cecum
p= 0.636
E.
Procrustes – Proximal colon
p= 0.526
F.
Procrustes – Distal colon
p= 0.037
